# CCL19 and CCL28 Assist Herpes Simplex Virus 2 Glycoprotein D To Induce Protective Systemic Immunity against Genital Viral Challenge

**DOI:** 10.1128/mSphere.00058-21

**Published:** 2021-04-28

**Authors:** Yan Yan, Kai Hu, Ming Fu, Xu Deng, Sukun Luo, Lina Tong, Xinmeng Guan, Siyi He, Chang Li, Wei Jin, Tao Du, Zifeng Zheng, Mudan Zhang, Yalan Liu, Qinxue Hu

**Affiliations:** a Center of Clinical Laboratory, The Fifth People's Hospital of Wuxi, Affiliated Hospital of Jiangnan University, Wuxi, China; b State Key Laboratory of Virology, Wuhan Institute of Virology, Center for Biosafety Mega-Science, Chinese Academy of Sciences, Wuhan, China; c Department of Gastroenterology, Guangzhou Women and Children's Medical Center, Guangzhou Medical University, Guangzhou, China; d Institute for Infection and Immunity, St. George’s, University of London, London, United Kingdom; U.S. Food and Drug Administration

**Keywords:** CCL19, CCL28, HSV-2, glycoprotein D, mucosal immunity

## Abstract

Potent systemic immunity is important for recalled mucosal immune responses, but in the defense against mucosal viral infections, it usually remains low at mucosal sites. Based on our previous findings that enhanced immune responses can be achieved by immunization with an immunogen in combination with a molecular adjuvant, here we designed chemokine-antigen (Ag) fusion constructs (CCL19- or CCL28-herpes simplex virus 2 glycoprotein D [HSV-2 gD]). After intramuscular (i.m.) immunization with different DNA vaccines in a prime and boost strategy, BALB/c mice were challenged with a lethal dose of HSV-2 through the genital tract. Ag-specific immune responses and chemokine receptor-specific lymphocytes were analyzed to determine the effects of CCL19 and CCL28 in strengthening humoral and cellular immunity. Both CCL19 and CCL28 were efficient in inducing long-lasting HSV-2 gD-specific systemic immunity. Compared to CCL19, less CCL28 was required to elicit HSV-2 gD-specific serum IgA responses, Th1- and Th2-like responses of immunoglobulin (Ig) subclasses and cytokines, and CCR3^+^ T cell enrichment (>8.5-fold) in spleens. These findings together demonstrate that CCL28 tends to assist an immunogen to induce more potently protective immunity than CCL19. This work provides information for the application potential of a promising vaccination strategy against mucosal infections caused by HSV-2 and other sexually transmitted viruses.

**IMPORTANCE** An effective HSV-2 vaccine should induce antigen (Ag)-specific immune responses against viral mucosal infection. This study reveals that chemokine CCL19 or CCL28 enhanced HSV-2 glycoprotein D ectodomain (gD-306aa)-induced immune responses against vaginal virus challenge. In addition to eliciting robust humoral immune responses, the chemokine-Ag fusion construct also induced Th1- and Th2-like immune responses characterized by the secretion of multiple Ig subclasses and cytokines that were able to be recalled after HSV-2 challenge, while CCL28 appeared to be more effective than CCL19 in promoting gD-elicited immune responses as well as the migration of T cells to secondary lymph tissues. Of importance, both CCL19 and CCL28 significantly facilitated gD to induce protective mucosal immune responses in the genital tract. The above-described findings together highlight the potential of CCL19 or CCL28 in combination with gD as a vaccination strategy to control HSV-2 infection.

## Abstract (precis)

An effective HSV-2 vaccine should induce antigen (Ag)-specific immune responses against viral mucosal infection. This study reveals that chemokine CCL19 or CCL28 enhanced HSV-2 glycoprotein D ectodomain (gD-306aa)-induced immune responses against vaginal virus challenge.

## INTRODUCTION

Genital herpes is a sexually transmitted disease. Although herpes simplex virus 1 (HSV-1) can cause genital herpes, most cases of genital herpes are caused by HSV-2, which infects the host mainly through the mucosa of the genital tract (1). More than 400 million individuals are infected by HSV-2 worldwide, resulting in significant global financial burden (1). HSV-2 replicates predominantly in the mucosal epithelial cells and establishes latency in the innervating neurons. HSV-2 infection can also increase the risk of HIV-1 transmission (2–4). Although protective vaccines against HSV-2 are persistently pursued and considerable efforts and expenditures have been made over the past several decades, such a vaccine remains elusive (5). For instance, an HSV-2 gD subunit vaccine combined with adjuvants consisting of 3-O-deacylated monophosphoryl lipid A and alum (MPL-alum) has not conferred significant efficacy against HSV-2 infection in the population (6). The genital tract and the rectal mucosae are portals of entry for sexually transmitted infections (STIs); however, they are immunologically restrictive tissues that prevent entry of activated and systemic memory T cells (7). Therefore, developing a vaccine against HSV-2 at the mucosal portals of entry could be the ideal strategy to control and prevent the disease.

CCL19 and CCL28, as molecular adjuvants, were recently described as potent elements for promoting cellular immune responses in systemic circulation and the mucosae, based on their potential impacts on T- and B-cell activation (8–11). CCL19 is one of the key molecules involved in establishing functional microenvironments for the initiation of immune responses in secondary lymphoid tissues (12, 13). CCL19, through interactions with its cognate receptor CCR7, plays a pivotal role in recruiting responsive immunocytes, including antigen (Ag)-specific or nonspecific T cells, B cells, and matured dendritic cells (DCs) (13, 14), to secondary lymphoid organs. CCL19 as a molecular adjuvant has been proven to be useful in several immunization models and is capable of promoting Ag-specific humoral and cellular immune responses in combination with vaccine candidates, including HSV-1 gB (15), HIV-1 Env (8), pseudorabies virus gB (16), and hepatitis C virus (HCV) core DNA (17). CCL28 is widely expressed in mouse, human, and pig mucosal tissues, including the mammary gland, salivary gland, small and large intestines, and trachea, where it appears to be predominantly produced by epithelial cells (18). CCL28 can bind to CCR3 and CCR10 receptors and plays essential roles in both innate and adaptive immunity, primarily by recruiting leukocytes to secondary lymphoid tissues and by extravasation of IgA antibody (Ab)-secreting cells (IgA ASCs) to mucosal surfaces in the gut and lactating mammary gland (18). The role of chemokines as adjuvants in inducing mucosal immune responses has been extensively assessed in HIV-1 (8, 11, 19, 20) and influenza A (21–24). In addition, these mucosal immunity-associated chemokines have been evaluated in helping lymphocytes to release antiviral-related cytokines, such as gamma interferon (IFN-γ) and interleukin-4 (IL-4), which can further promote T cell proliferation and Ag uptake by DCs (8, 25). However, the mechanisms of and differences between CCL19 and CCL28 as adjuvants in promoting HSV-2 glycoprotein D (gD)-specific mucosal immunity, especially protecting animals from intravaginal (i.vag.) virus challenge, need further exploration, which may facilitate the development of more potent, durable, and safe T cell-based antiviral pharmaceuticals or vaccines.

Using mice as a vaccination and challenge model, we searched for approaches to improve HSV-2 glycoprotein immunogenicity. For comparison purposes, we first produced bicistronic- and chimeric-expressing DNA vectors using an internal ribosome entry site (IRES)- and a GCN4-based isoleucine zipper trimerization domain (IZ) linker, respectively. The GCN4-based linker allowed us to develop stabilized native-like multimers and facilitated the coexpression of Ags and chemokines (26, 27). We further compared the capability of CCL19 and CCL28 in assisting an immunogen to induce protective immune responses against HSV-2 infection.

## RESULTS

### Construction and characterization of bicistronic- and IZ-based chemokine-Ag DNA vectors.

**FIG 1:**
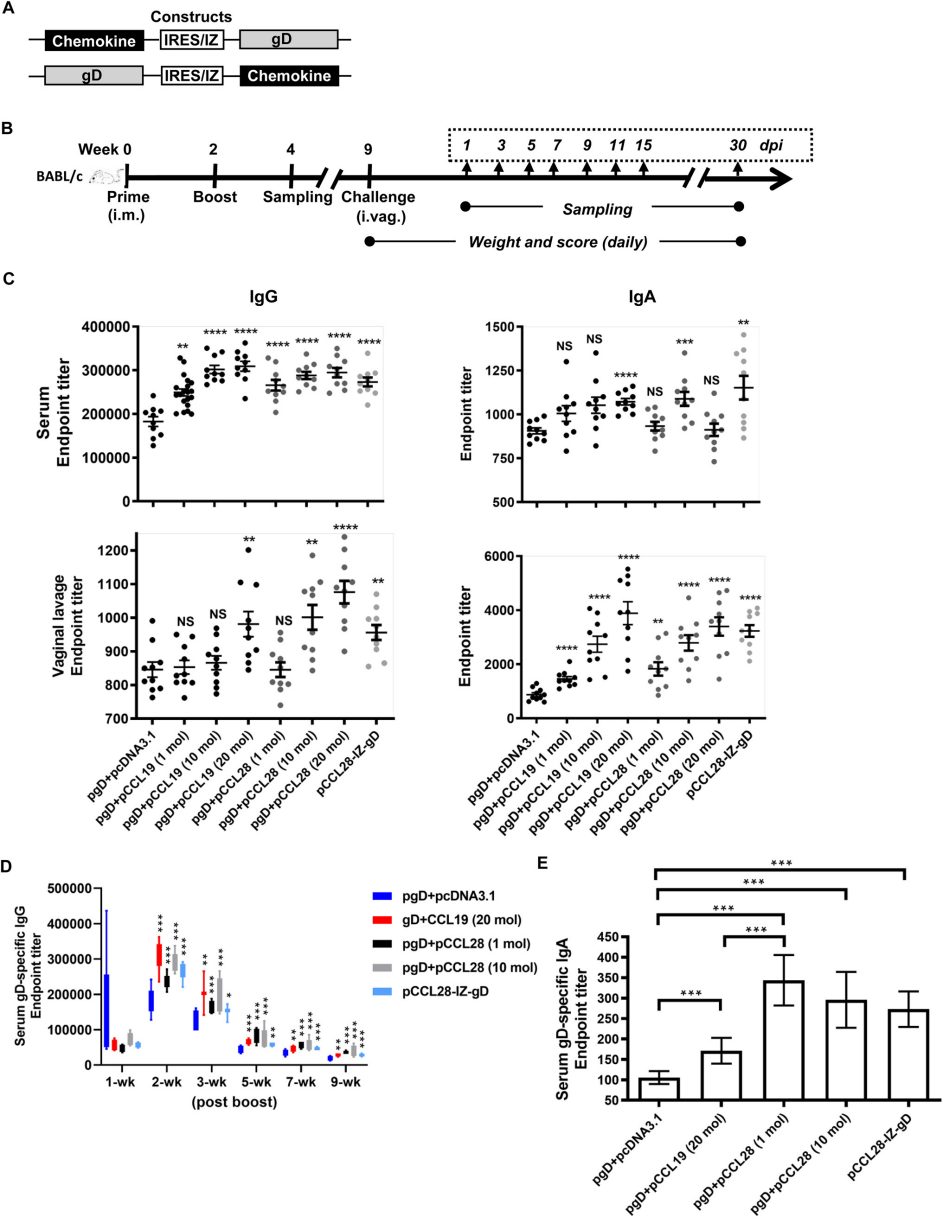
Construct design, immunization procedures, and humoral immune responses of sera and vaginal lavage fluid. (A) Schematics of chemokine-linker-gD and gD-linker-chemokine constructs. (B) Schedule of i.m. immunization and i.vag. challenge. Mice (*n* = 10) were immunized twice with pcDNA3.1, pgD + pcDNA3.1, pgD + pCCL19, or pCCL28 chemokine-Ag plasmids in saline solution at 0 and 2 weeks. Two weeks postboost, mice (*n* = 5) in each group were sacrificed for tissue collection, while the rest were used for the challenge experiments. Postchallenge, the weight and clinical symptoms of all mice were monitored every day for 15 days. Murine sera, vaginal lavage, and sacral ganglia were collected at the indicated time points for subsequent tests. (C) Humoral immune responses induced by pgD in combination with pCCL19 or pCCL28 and pCCL28-IZ-gD. Mice were immunized i.m. at 0 and 2 weeks by electroporation. At 2 weeks (wk) postboost, sera and vaginal lavage fluid were collected and an endpoint titration of gD-specific IgG and IgA was measured by ELISA. Data are the means ± SEM, *n* = 10 mice per group, from at least two independent experiments with each condition being performed in duplicate. (D) Serum gD-specific IgG was compared for each group at the indicated weeks postboost. (E) Serum gD-specific IgA at 7 weeks postboost was compared for each group. Data shown are the means ± SEM (*n *=* *5 mice/group) from three independent experiments with each condition performed in duplicate. Statistically significant differences were determined by comparison to the pgD + pcDNA3.1 group. NS, not statistically significant; *, *P < *0.05; **, *P* < 0.01; ***, *P < *0.001; ****, *P < *0.0001.

Bicistronic- or IZ-based chemokine-Ag DNA plasmids were designed to ensure the coexpression of HSV-2 gD with chemokine CCL19 or CCL28 within the same cell (Fig. 1A; see also Tables S1 and S2 and Fig. S1A in the supplemental material). The effects of IRES- and IZ-based DNA vaccines were compared with Ag DNA and corresponding chemokine DNA in combination *in vivo*. We first compared the immunogenicity of constructs with CCL19 or CCL28 on the upstream or downstream of HSV-2 gD. Following immunization and animal protection study, we found that pgD-IRES/IZ-CCL19 and pCCL28-IRES/IZ-gD induced better immune responses (Table S3, Fig. S1B). Among all the tested fusion constructs, pCCL28-IZ-gD induced the best protection immunity in mice, with a symptom score of zero after HSV-2 challenge. Therefore, we selected this construct for the downstream study. As evidenced by Western blotting, the gD portion of the pCCL28-IZ-gD fusion protein construct was successfully expressed in the cell line to a level similar to that of the gD protein as a dimer (Fig. S1C). Further Western blot and enzyme-linked immunosorbent assay (ELISA) analyses showed that the proteins of gD, CCL19, and CCL28 in the supernatants of transfected HEK 293T cells (*in vitro*) and immunized mice (*in vivo*) were correctly expressed (Fig. S1B and S1D). Notably, due to the different standard products in the CCL19 and CCL28 ELISA kits, the chemokines displayed distinct expression (Fig. S1D).

10.1128/mSphere.00058-21.1 TABLE S1 Sequences of primers and digestion sites of “chemokine-IRES-gD” and “gD-IRES-chemokine” bicistronic expression constructs. Download Table S1, DOCX file, 0.02 MB. Copyright © 2021 Yan et al. 2021 Yan et al. https://creativecommons.org/licenses/by/4.0/ This content is distributed under the terms of the Creative Commons Attribution 4.0 International license.

10.1128/mSphere.00058-21.2 TABLE S2 Sequences of primers and digestion sites of “chemokine-IZ-gD” and “gD-IZ-chemokine” fusion constructions. Download Table S2, DOCX file, 0.02 MB. Copyright © 2021 Yan et al. 2021 Yan et al. https://creativecommons.org/licenses/by/4.0/ This content is distributed under the terms of the Creative Commons Attribution 4.0 International license.

10.1128/mSphere.00058-21.3 TABLE S3 HSV-2 challenge and score after HSV-2 challenge. Download Table S3, DOCX file, 0.02 MB. Copyright © 2021 Yan et al. 2021 Yan et al. https://creativecommons.org/licenses/by/4.0/ This content is distributed under the terms of the Creative Commons Attribution 4.0 International license.

10.1128/mSphere.00058-21.4 FIG S1 Characterizations of the DNA constructs expression and responses in vaccinated mice. (A) pgD-IZ-CCL19 fusion construct in plasmid pcDNA3.1(+). IZ linker was amplified by splicing overlap extension PCR and pgD-IZ-CCL19 gene was cut and ligated into the plasmid vector pcDNA3.1(+). (B) HSV-2 gD-specific IgG endpoint titer for each vaccinated group. Antisera were collected at 2 weeks postboost and used for the detection of HSV-2 gD-specific IgG endpoint titrations for each vaccinated group by direct ELISA. Error bars represent means ± SEM, *n* = 5 mice per group, and at least two independent experiments with each condition performed in duplicate. Statistically significant differences determined by comparing to the pgD plus pcDNA3.1 group are indicated. (C) gD expression of gD and CCL28-linker-gD chimeric constructs. For testing Ag expression, HEK 293T cell monolayers in 12-well plates were transfected with 1 mol of plasmids (*in vitro*), using Lipofectamine 2000 (Invitrogen) according to the manufacturer’s instructions. After 48 h, supernatants were harvested and cells were pelleted (10 min, 2,000 × g at 4°C). The immunized sera at 2 weeks postboost (*in vivo*) were collected and gD expression was analyzed by SDS-PAGE and native PAGE, followed by Western blotting. (D) Expression of the chemokines CCL19 and CCL28 *in vitro* and *in vivo*. The chemokine CCL19 or CCL28 concentrations of the supernatants from transfected cells (*in vitro*) or sera (*in vivo*) were quantified by DuoSet ELISA kit. Data are the means ± SEM, *n* = 5 mice per group and at least three independent experiments were performed in triplicated for each condition. (E) Analysis of CCL19 or CCL28 receptor expression on Ag-responsive splenocytes in vaccinated and challenged mice. Murine splenocytes were separated at 2 weeks postboost and 9 days postinfection (dpi) and analyzed by flow cytometry. Representative flow cytometric data showing the percentages of CCR7^+/−^ or CCR10^+/−^ B (CD19^+^), T (CD3^+^), and DC (CD11c^+^)-cell in whole splenocytes of indicated groups. Data are representative three independent experiments. CCL19, 20 mol; CCL28, 10 mol. Statistically significant differences between the groups are indicated. NS, not statistically significant; *, *P* < 0.05; **, *P* < 0.01; ***, *P < *0.001. Download FIG S1, PDF file, 0.3 MB. Copyright © 2021 Yan et al. 2021 Yan et al. https://creativecommons.org/licenses/by/4.0/ This content is distributed under the terms of the Creative Commons Attribution 4.0 International license.

### CCL19 and CCL28 are potent in assisting HSV-2 gD to induce long-lasting humoral responses postimmunization.

We have previously found that CCL19 enhances the immunogenicity of HSV-2 gB (28), but little is known about the role and the mechanism of CCL28 in promoting antiviral responses during reproductive tract infection. After immunization of all plasmid groups in BALB/c mice (Fig. 1A and B, Table S3), we compared the gD-specific IgG in murine sera at 2 weeks postboost of all groups (Fig. S1B) and the acute disease severities after HSV-2 challenge (Table S3). The results showed that only pgD plus pCCL19 (10 mol and 20 mol), pgD plus pCCL28 (1 mol, 10 mol, and 20 mol), and pCCL28-IZ-gD (1 mol) groups scored zero for symptom severity during the observational days postinfection (dpi) (Table S3). As shown in Fig. 1C, mice coimmunized with 1 mol pgD plus 20 mol pCCL19 or 10 mol pCCL28, or 1 mol pCCL28-IZ-gD fusion construct, significantly enhanced the levels of gD-specific IgG and IgA titers, compared to the pgD plus pcDNA3.1 group in both sera and vaginal lavage fluid at 2 weeks postboost. Notably, we observed that mice coimmunized with more CCL28 (20 mol) had ruffled hair and low level of serum IgA but a high level of mucosal IgA, indicating that 20 mol pCCL28 injection caused some side effects and difference of IgA expression in blood and mucosae. Altogether, the amount of CCL28 required may be less than that required for CCL19 to induce a similar level of effective humoral immunity. Additionally, the chimeric construct pCCL28-IZ-gD showed a distinct ability to enhance gD-specific systemic and mucosal Ab responses.

The duration and quality of humoral immunity and the generation of immunological memory are critical for generating protective immunity. To investigate whether gD coimmunized with mucosa-associated chemokines could induce potent and long-lasting humoral immunity postimmunization and postchallenge, mice were coimmunized with gD and chemokines and then challenged with lethal HSV-2 at 9 weeks postboost. Murine sera were collected for gD-specific IgG and IgA titrations postboost and postchallenge for several weeks as indicated in Fig. 1B. As shown in Fig. 1D, all chemokine coimmunized mice displayed enhanced titers of gD-specific IgG until 9 weeks postboost compared with the responses of the pgD plus pcDNA3.1 group. Compared with the pgD plus 1 mol pCCL28 group, mice immunized with pCCL28-IZ-gD developed a similar magnitude of antibody (Ab) response. For the detection of serum gD-specific IgA at 7 weeks postboost, gD coimmunized with CCL28 (1 mol or 10 mol pCCL28 or the pCCL28-IZ-gD chimeric construct) showed relatively higher mucosal Ab titers (IgA) than pgD plus 20 mol pCCL19 (almost 2-fold increased; Fig. 1E). Here, CCL28 is more effective than CCL19 in promoting long-lasting mucosal immunity postimmunization.

### CCL19 and CCL28 are potent in assisting HSV-2 gD to induce a robust humoral response and overall magnitude of gD-specific Ig subclasses in serum postchallenge.

**FIG 2:**
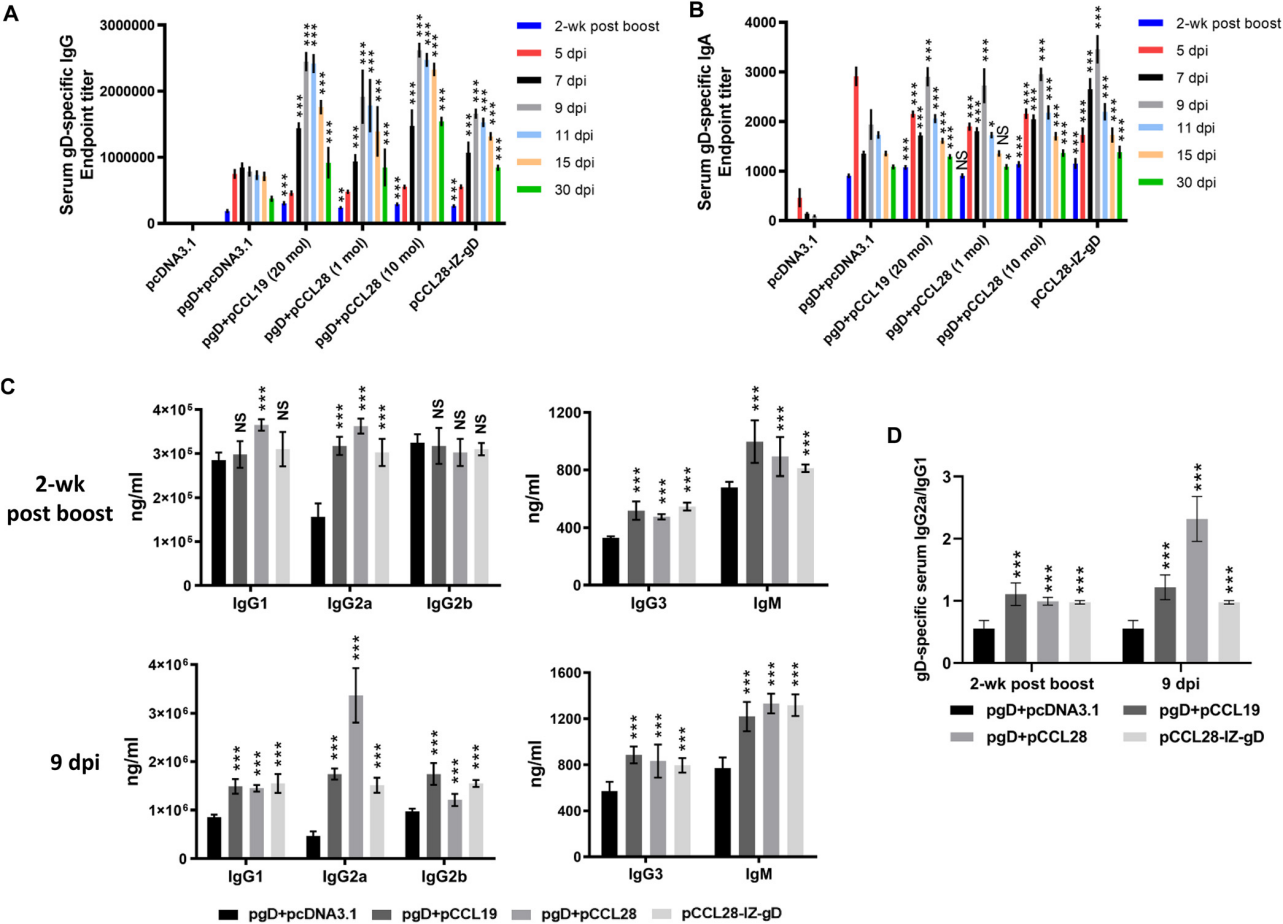
gD-specific IgG and IgA responses and Ig subclasses in serum postchallenge. Serum gD-specific IgG (A) and IgA (B) at indicated days postboost and postchallenge. The endpoint titrations of gD-specific IgG and IgA were determined by ELISA separately. (C) The distribution of gD-specific IgG1, IgG2a, IgG2b, IgG3, and IgM isotypes in murine sera at 2 weeks postboost (upper) and 9 dpi (lower). (D) gD-specific IgG2a/IgG1 ratio of the different groups at 2 weeks postboost and 9 dpi. The quantification of each Ig subclass was measured by ELISA and calculated according to the standard curve obtained using corresponding Ig subclass standards. CCL19, 20 mol; CCL28, 10 mol. Data are the means ± SEM, *n* = 5 or 7 mice per group, from at least two independent experiments with each condition being performed in duplicate. Statistically significant differences determined by comparison to the pgD + pcDNA3.1 group are indicated. NS, not statistically significant; *, *P < *0.05; **, *P* < 0.01; ***, *P < *0.001.

Post-HSV-2 challenge, there was a nearly 2-fold increase in the recall of serum gD-specific IgG in most of the chemokine-coimmunized groups compared with pgD plus pcDNA3.1 (Fig. 2A). Of note, the pgD plus pcDNA3.1 group had an initial increase in humoral gD-specific IgG and IgA titer at 5 dpi, and the highest level of responding Ab in the chemokine coimmunized groups appeared at 9 dpi and remained up to 30 dpi, whereas antibody titers of the experimental groups with good protection effects decreased gradually (Fig. 2A and B). In particular, the pCCL28-IZ-gD group developed higher levels of gD-specific IgG and IgA compared to pgD plus pCCL28 (1 mol) at 7 dpi and 9 dpi, respectively. In general, coimmunization with gD and CCL28 plasmid showed a comparable ability to enhance the duration of Ag-specific IgG and IgA in sera postboost and postchallenge. In contrast, the Ab titer results suggested that the chimeric construct was more stable and effective than a 1:1 mixture of pgD with pCCL28; the other deserted vaccinated groups did not all produce such high levels of recalled Ab titers (data not shown). Therefore, in the following studies we used 1 mol pgD mixed with 20 mol pCCL19 or 10 mol pCCL28 and 1 mol pCCL28-IZ-gD.

It has been previously described that mice immunized with a plasmid encoding HSV-2 gD had a substantial level of specific IgG in their sera, which was almost exclusively of the IgG1 isotype and not IgG2a or IgG2b (29, 30). To test whether the pCCL19 or pCCL28 chemokine simply mixed with pgD was functional in modulating the isotypes of the antibody responses, we examined the levels of gD-specific IgG subtypes (IgG1, IgG2a, IgG2b, and IgG3) and IgM by ELISA in sera collected at 2 weeks postboost and 9 days postchallenge. The IgG2a/IgG1 ratio was used as a surrogate marker of Th1 and Th2 responses (27). Here, we measured the gD-specific IgG2a/IgG1 ratio to evaluate the type of Th response induced by our vaccine formulations. Among the four IgG subtypes in mice, only IgG1 is associated with a Th2 profile (31). Among the four DNA vaccines, mice immunized with pgD plus 10 mol pCCL28 developed significantly augmented Th1 and Th2 responses (IgG1, IgG2a, IgG3, and IgM) compared with pgD plus pcDNA3.1 at 2 weeks postboost, whereas other groups only enhanced Th1 subclass responses (IgG2a, IgG3, and IgM) (Fig. 2C, upper). However, all groups switched to enhance all Ig subclass responses after HSV-2 i.vag. challenge (9 dpi), and the magnitude of the gD-specific IgG2a subclass was significantly augmented in the pgD plus pCCL28 group (Fig. 2C, lower). As shown in Fig. 2D, the CCL19 (20 mol) and CCL28 (1 mol and 10 mol) chemokines all helped to augment Th1-biased Ig subclass responses compared with pgD plus pcDNA3.1; notably, pgD coimmunized with 10 mol pCCL28 showed a dramatic increase (nearly 5-fold) in the Th1-biased response compared with pgD plus pcDNA3.1 at 9 dpi. Taken together, these results suggest that pgD coimmunized with higher doses of pCCL28 (10 mol) significantly augmented the Th1-biased gD-specific Ig subclasses in recalled responses compared with pgD plus pCCL19 and pgD plus pcDNA3.1, while CCL28 and gD used in a chimeric construct is capable of augmenting gD-specific antibody responses toward a balanced Th1 and Th2 profile in protective immune responses.

### CCL19 and CCL28 are potent in assisting HSV-2 gD to induce neutralizing responses in sera and vaginal lavage fluids.

**FIG 3:**
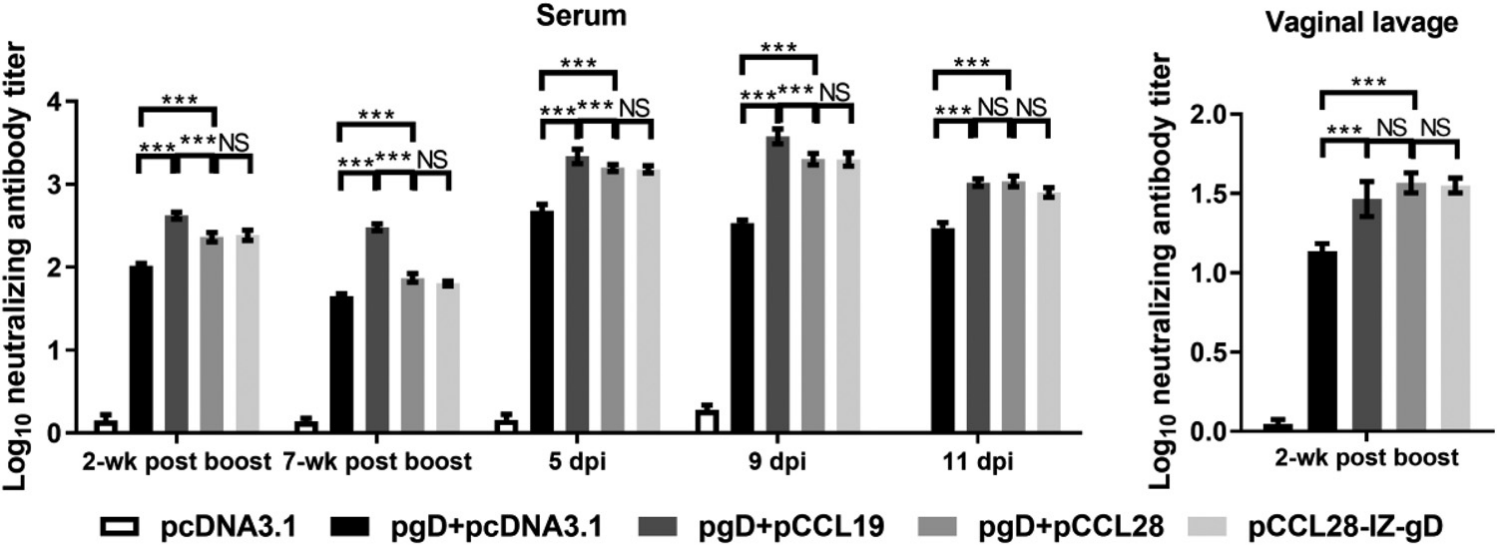
Anti-HSV-2-neutralizing activities of sera and vaginal lavage fluid from immunized mice. Murine sera collected at the indicated times were tested at dilutions starting with 1:10 (at 2 weeks and 7 weeks postboost) and 1:40 (at 5, 9, and 11 dpi). Vaginal lavage fluid collected at 2 weeks postboost were tested at dilutions starting with 1:5. Plaque numbers were determined for each sample and plotted as percent inhibition. NS, not statistically significant; ***, *P < *0.001.

The results described above demonstrated that pgD coimmunized with pCCL19 or pCCL28 plasmids or a pCCL28-IZ-gD chimeric construct can significantly augment the magnitudes of systemic and mucosal Ab responses and their subclasses to gD. There is well-documented evidence in animal models that an efficacious prophylactic HSV-2 vaccine will most likely induce a robust neutralizing antibody response, especially at mucosal sites (2, 32, 33). We next assessed whether such increases in antibodies correlated with concomitant increases in viral neutralizing responses, especially post-HSV-2 challenge. The neutralizing activities of sera collected at 2 weeks and 7 weeks postboost and at 5, 9, and 11 dpi, and vaginal lavage fluid at 2 weeks postboost were tested. As shown in Fig. 3, neutralizing activities of sera and vaginal lavage fluid against HSV-2 were significantly enhanced at the indicated time points in mice immunized with pgD plus pCCL19 or CCL28 or the pCCL28-IZ-gD chimeric construct compared to the pgD plus pcDNA3.1 group. Notably, the neutralizing activity of sera remained apparent in the pgD plus pCCL19 group up to 7 weeks postboost and appeared to remain at a higher level than the CCL28 group before 9 dpi, but the neutralizing antibody in the mucosae showed no advantage. In addition, the neutralizing activity peaked at 9 dpi and remained constant in the CCL28 group up to 11 dpi. Taken together, the neutralizing activity of sera from mice immunized with pgD plus pcDNA3.1 was significantly lower than that of mice coimmunized with gD and chemokines. Our results suggest that both CCL19 and CCL28 tend to assist an immunogen to induce more sustainable and potent neutralizing antibody responses than the pgD plus pcDNA3.1 group postchallenge.

### CCL19 and CCL28 are potent in assisting HSV-2 gD to promote Ag-specific Th1 and Th2 cellular immune responses.

**FIG 4:**
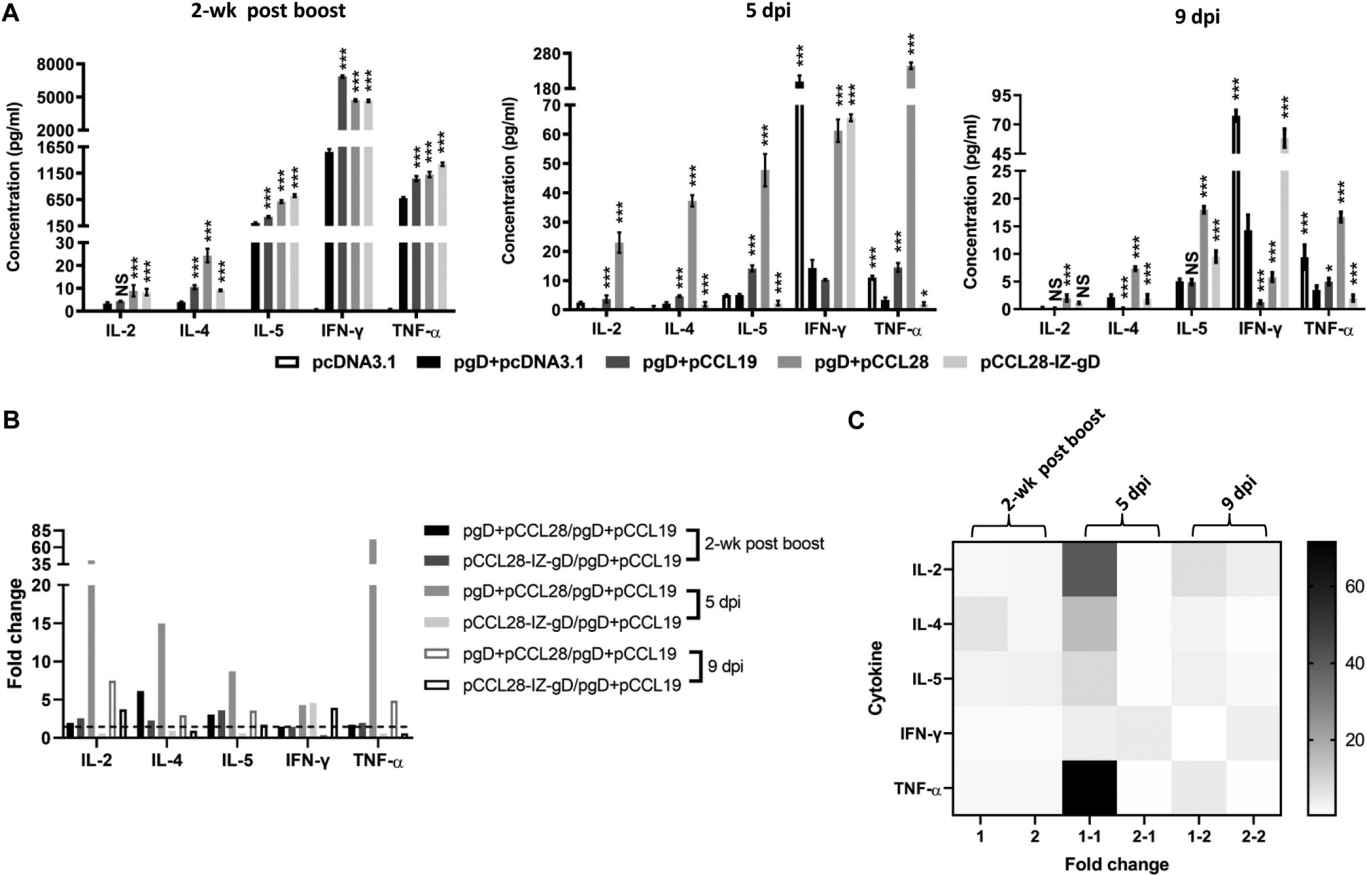
Ag-specific Th1/Th2-associated cytokine production from splenocytes and antisera. (A) Cytokine production in splenocyte supernatants from immunized mice (2 weeks postboost) and antisera after HSV-2 challenge (5 dpi and 9 dpi) was quantified by flow cytometry. (B) Fold changes in the pgD + pCCL28 or pCCL28-IZ-gD groups over the pgD + pCCL19 group. Splenocytes (1 × 10^7^ cells) isolated from all groups postboost were stimulated with purified gD (2 μg/ml) and cocultivated for 5 days, and then the supernatants were collected. (C) Hot map of cytokine fold changes (i.e., pgD + CCL28/pgD + CCL19 and pCCL28-IZ-gD/pgD + CCL19). Group 1, 1-1, and 1-2 is named pgD + CCL28/pgD + CCL19, and 2, 2-1, and 2-2 is named pCCL28-IZ-gD/pgD + CCL19. Antisera were collected at 5 and 9 dpi. The production of Th1 (IL-2, IFN-γ, and TNF-α)- and Th2 (IL-4 and IL-5)-associated cytokines was detected using a CBA kit. Data shown are representative of the mean cytokine concentrations (pg/ml) ± SEM with *n* = 5 mice per group. Statistically significant differences determined by comparing to the pgD + pcDNA3.1 group are indicated. NS, not statistically significant; *, *P < *0.05; ***, *P < *0.001.

It has been suggested that CCL19 and CCL28, as key regulators *in vivo*, regulate T cell and DC-dependent adaptive immune responses, resulting in lymphoproliferative responses and indirectly inducing B cell upregulation (8, 17). We used the same concept to speculate that codelivery of pgD with the pCCL19 or pCCL28 plasmid can target and activate Ag-specific immunocytes and produce balanced Th1- and Th2-like cellular immune responses to substantially improve the protective effects of the DNA vaccine. It is generally known that IL-2, tumor necrosis factor alpha (TNF-α), and IFN-γ are associated with a Th1 profile, whereas IL-4 and IL-5 are associated with a Th2 profile. Given the central roles of cytokines secreted by activated immunocytes in defining the subsequent immune responses, we next evaluated Ag-specific Th1- or Th2-like cellular immune responses by measuring the associated cytokines secreted by murine splenocytes postboost and postchallenge in response to gD protein stimulation. As shown in Fig. 4A, splenocytes of mice coimmunized with pgD (1 mol) and pCCL19 (20 mol) or pCCL28 (10 mol) or the pCCL28-IZ-gD (1 mol) chimeric construct produced enhanced levels of Th1- and Th2-associated cytokines compared with pgD plus pcDNA3.1 after HSV-2 gD stimulation *in vitro* postboost. However, compared with the pgD plus pcDNA3.1 group, the pgD plus pCCL19 group induced relatively higher levels of cytokines at 5 dpi, although it was lower than that in mice coimmunized with pgD plus pCCL28 or pCCL28-IZ-gD. Interestingly, the pgD plus pCCL28 group developed higher levels of overall Ag-specific Th1- and Th2-like cytokines post-HSV-2 challenge, while the pCCL28-IZ-gD chimeric construct developed higher levels of IL-2, IL-5, and IFN-γ at 9 dpi than the pgD plus pCCL19 group (Fig. 4B and C). In contrast, supernatants from the negative-control group (pcDNA3.1) demonstrated that IL-2, IL-4, and IL-5 levels were below the limits of detection postboost, while there were significantly higher levels of inflammation-related cytokines IFN-γ and TNF-α at 5 dpi and 9 dpi in antisera compared with the pgD plus pcDNA3.1 group, consistent with their disease severity (Fig. 4A); we speculated that this was associated with the expression of viral immune evasion genes and the inflammatory response to virus-infected cells (severe and critical inflammation was observed in mice). Collectively, these results indicate that codelivery of CCL28 DNA vaccines is capable of strengthening the magnitude and duration (up to 9 dpi) of Th1- and Th2-like Ag-specific cellular responses in protective immune responses.

### CCL19 and CCL28 are potent in chemoattracting Ag-responding T cells to secondary lymph nodes.

**FIG 5:**
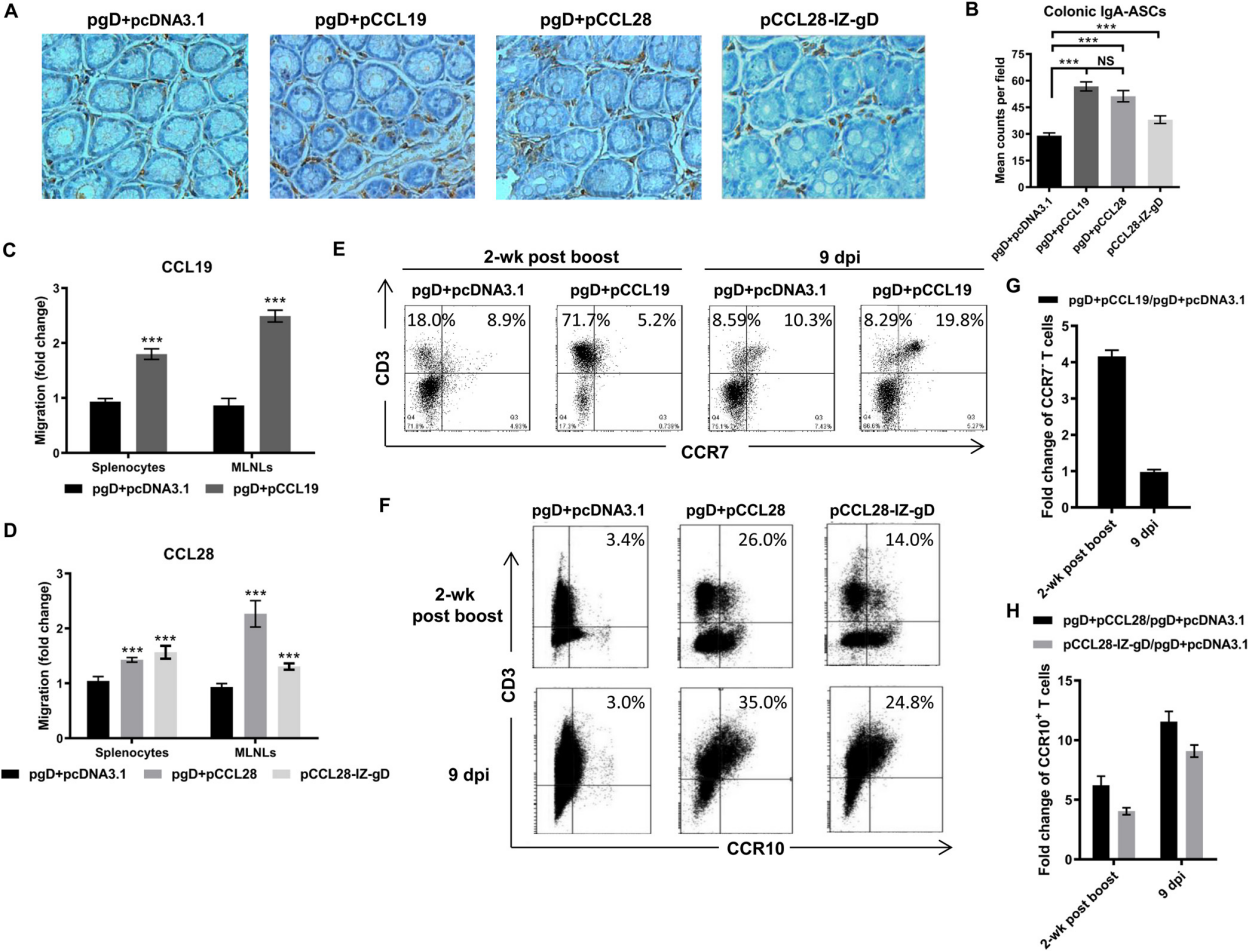
Responsive immunocyte migration to secondary lymph nodes postimmunization and postchallenge. IgA^+^ cells at colorectal sites mediated by the CCL19 or CCL28 adjuvant in immunized mice. (A) IgA^+^ cells in murine colorectal mucosal samples were stained with DBA and hematoxylin. Data shown are representative immunohistochemistry results (magnification, ×200). (B) Mean counts of IgA^+^ cells of 10 high-power fields for each group. Migration of murine splenocytes and MLNLs for each group in response to murine CCL19 or CCL28 protein. Single lymphocytes were prepared and counted for the chemotactic response to CCL19 or CCL28 using a Transwell system, and the fold changes were calculated compared to the cell number in the lower chamber without CCL19 (C) or CCL28 (D). The frequencies of CCR7^+/−^ (E) and CCR3^+^ (F) CD3^+^ splenocytes at 2 weeks postboost and 9 dpi were analyzed by flow cytometry. By comparing to the pgD + pcDNA3.1 group, the fold change of CCR7^−^ (G) and CCR3^+^ (H) CD3^+^ splenocyte frequencies in the pCCL19 or pCCL28 adjuvant groups were calculated, and the gating strategies are shown in Fig. S1E. Data shown are the means ± SEM for each group (*n* = 5 mice/group). Statistically significant differences determined by comparing to the pgD + pcDNA3.1 group are indicated. NS, not statistically significant; ***, *P < *0.001.

Following the demonstration that codelivery of pgD (1 mol) and pCCL19 (20 mol) or pCCL28 (10 mol) or the pCCL28-IZ-gD (1 mol) chimeric construct could enhance gD-specific systemic and mucosal antibody responses as well as Th1- and Th2-like cellular responses, we next investigated the underlying mechanisms of such an immune enhancement. We previously demonstrated that CCL19 fused with HSV-2 gB constructs could induce a responsive immunocyte increase in murine secondary lymph nodes and at mucosal sites postimmunization (28). In contrast, CCL28 remains poorly understood, especially with regard to its performance after virus challenge. It is widely known that CCL19 and CCL28 are involved in trafficking the CCR7^+^ and CCR3/CCR10^+^ immunocytes, including IgA^+^ plasma cells, into colorectal mucosal sites, (8, 19, 20, 31), but their promotional effects may be different during immune protection. According to the role of IgA^+^ cells in the adaptive immune responses to pathogens at mucosal sites, our immunohistochemistry results also showed that pgD coimmunized with pCCL19 or pCCL28 or the pCCL28-IZ-gD chimeric construct developed increased IgA^+^ cells at a number of rectal sites (Fig. 5A); their cell numbers were increased up to nearly 1.8-fold compared with the pgD plus pcDNA3.1 group (Fig. 5B). Notably, these results coincided with gD-specific IgA titer changes in vaginal lavage fluid. In summary, codelivery of pCCL19 or pCCL28 with pgD show comparable ability in attracting IgA^+^ cells to colorectal mucosal sites, reflecting enhanced mucosal immune responses.

Similarly, spleen and mesenteric lymph nodes (MLNs) are important secondary lymph nodes and mucosal sites, respectively, and they are associated with lymphocyte homing and recycling in mice. As shown in Fig. 5C and D, these two chemokines effectively induced the chemotaxis and migration of their responsive splenocytes and MLN lymphocytes (MLNLs). Compared with pgD plus pcDNA3.1, mice coimmunized with pgD plus pCCL19 or pCCL28 plasmids resulted in ∼1.8- and 1.4-fold increases, respectively, in the number of chemokine-responsive immunocytes that migrated to the spleen, and ∼2.6- and 2.4-fold increases, respectively, in the MLNs. In contrast, there was a slight decrease in the MLNs with the pCCL28-IZ-gD group. In general, codelivery of either pCCL19 or pCCL28 with pgD consistently resulted in higher levels of responsive immunocyte influx into the spleen and MLNs, but the MLNL response was higher.

**FIG 6:**
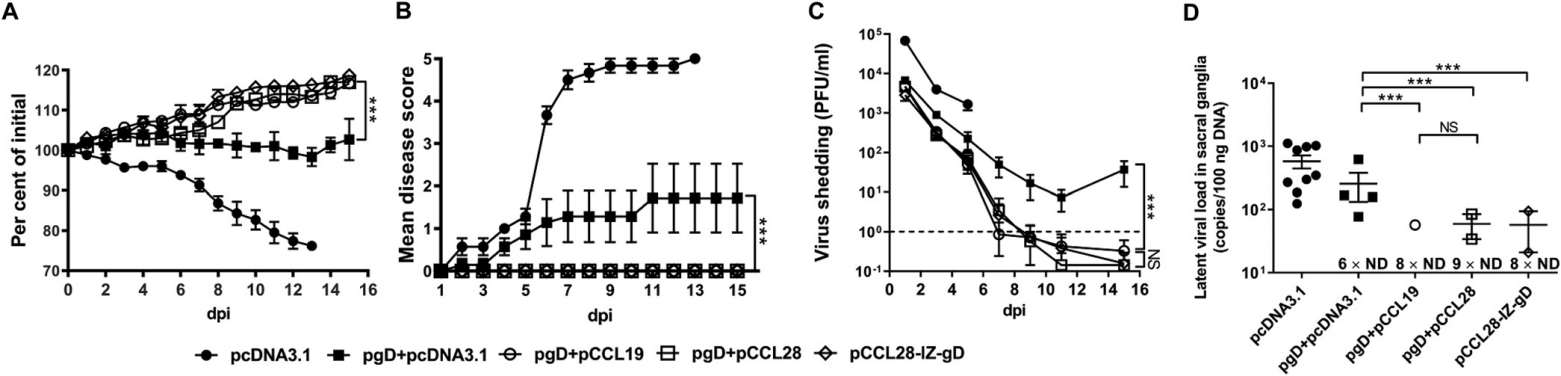
Protective efficacies of vaccine formulations during lethal vaginal HSV-2 challenge. Nine weeks postboost, mice were challenged i.vag. with a lethal dose of HSV-2. Vaccinated and control groups were monitored for up to 15 days for weight changes, illness features, and mortality. The figure shows weight changes (A), disease severity (B), viral shedding in vaginal lavage fluid (C), and latent viral DNA loads (D). Viral shedding was detected by plaque assay at the indicated time points. The dashed line indicates the detection limit. Latent viral DNA in the sacral ganglia was quantified by real-time PCR at 30 dpi or the date of death. The values 6 × ND, 8 × ND, and 9 × ND indicate the number of mice without latent virus. Data shown are the means ± SEM for each group (*n *=* *5 or 7 mice/group) from at least two independent experiments with each condition performed in duplicate. Statistically significant differences determined by comparing to the pgD + pcDNA3.1 group are indicated. NS, not statistically significant; ***, *P < *0.001. ND, no virus detected.

To better understand the performance of CCL19- or CCL28-mediated cellular immunity during challenge, we conducted assays to determine if the Ag-responding T cells migrate to secondary lymph nodes after HSV-2 infection. CCR7 presents as a defining factor for nonpolarized central (CCR7^+^) and polarized effector memory (CCR7^−^) T cells (34). CCR7 is expressed at high levels on naive and central memory T cells and enables homeostasis T cell subsets to recirculate and home to T cell areas in lymphoid organs, such as the white pulp areas of the spleen and lymph nodes (35, 36). We detected CCR7^+/−^ or CCR3^+^ T cells among splenocytes by flow cytometry postboost and postchallenge. As shown in Fig. 5E and 6F, CCR7^+/−^ or CCR3^+^ T cell-sensitized lymphocytes increased significantly in spleens postchallenge. Mice coimmunized with pgD plus pCCL19 showed an ∼4-fold increase in CCR7^−^ T cells among splenocytes compared with the pgD plus pcDNA3.1 group postboost (Fig. 5G), but a significant increase of CCR7^+^ T cell was found in the pgD plus pCCL19 group postchallenge (∼1.7-fold at 9 dpi) (Fig. 5E), while coimmunization with pgD plus pCCL28 plasmids or the pCCL28-IZ-gD construct displayed up to ∼10.2-fold and ∼8.5-fold increases in CCR3^+^ T cells in spleens postboost and postchallenge (Fig. 5F and H), respectively. The advantage over CCL19 was that CCL28 developed consistent enhancement of CCR10^+^ on B cells (CD19^+^) and DC (CD11c^+^) in immunized and challenged mice (Fig. S1E). Similar responses were found in the MLNs (data not shown). Accordingly, these results suggested that codelivery of CCL28 is more effective than CCL19 in enhancing cellular responses during protective immune responses.

### CCL19 and CCL28 are potent in assisting HSV-2 gD to induce immune protection against viral challenge in genital tract.

The mouse models of HSV-2 infection have provided important insights into the immunobiology of genital herpes. Studies that measure the frequency and quantity of viral shedding detected from the genital tract have provided insights into the natural history and pathogenesis of HSV-2 infection. An alternative vaccination goal is to alter the natural history of this infection by decreasing the quantity of virus that establishes latency in neurons and minimizing the quantity of virus shed from genital surfaces (33). Several candidate vaccines appear to reduce the quantity of HSV-2 in mouse and guinea pig sacral nerves (2). We next tested whether the mice coimmunized with pgD (1 mol) and pCCL19 (20 mol) or pCCL28 (10 mol), or the pCCL28-IZ-gD (1 mol) construct, can establish an efficient immune defense against a lethal dose of HSV-2 challenge by various mechanisms, including by activating relevant immune cells, to reduce the morbidity and mortality. The immunized mice were challenged i.vag. with a lethal dose of HSV-2 at 9 weeks postboost. Following challenge, the weights and clinical symptoms of the mice were monitored daily up to 15 dpi. The blood, vaginal lavage fluid, and sacral ganglia were sampled at the indicated time points, and thereafter we investigated viral shedding and latent viral DNA loads postchallenge.

### (i) Weight change.

After HSV-2 challenge, mice immunized with pcDNA3.1 exhibited apparent weight loss at 1 dpi, followed by a sharper loss after 6 dpi. In contrast, no apparent weight loss was observed when mice were coimmunized with pgD plus pCCL19, pCCL28, or pCCL28-IZ-gD. In fact, a significant weight increase compared with pgD plus pcDNA3.1 was observed (Fig. 6A).

### (ii) Disease severity.

For all of the less effective groups, symptoms began to appear at 2 dpi. In line with previous findings of mice immunized with pcDNA3.1, they developed severe symptoms rapidly, and all were dead at 13 dpi (28). In contrast, almost 40% of mice immunized with pgD plus pcDNA3.1 also developed relatively mild symptoms 3 dpi (inflammation, genital ulceration, and hair loss). Meanwhile, unlike the other groups, mice coimmunized with pgD plus pCCL19, pCCL28, or the pCCL28-IZ-gD construct did not show apparent symptoms (Fig. 6B).

### (iii) Viral shedding.

Viral loads in vaginal lavage fluid were detectable but dropped sharply in all groups; however, viral loads were the highest in the negative-control group and moderately high in the pgD plus pcDNA3.1 group, and the symptoms in the genital tracts and nerves of the negative-control mice were too severe to sample at 6 dpi. All of the mice were dead at 13 dpi. In contrast, viral shedding at mucosal sites was sharply decreased in the pgD plus pCCL19 or pCCL28 and pCCL28-IZ-gD groups. Those mice all survived, and their viral loads in the vaginal lavage fluid dropped below the detection limit at 7 dpi. Of note, viral reshedding was observed at 15 dpi in the pgD plus pcDNA3.1 group (Fig. 6C). Similar viral shedding incidences were observed in mice postchallenge (Table 1).

**TABLE 1:** HSV-2 shedding incidences in HSV-2-challenged mice^*a*^

Group	Total no. of animals	No. (%) of mice challenged with HSV-2 and virus shedding incidences by day dpi						
		1	3	5	7	9	11	15
pcDNA3.1	14	14 (100.0)	14 (100.0)	14 (100.0)	—	—	—	—
pgD + pcDNA3.1	13	13 (100.0)	13 (100.0)	10 (76.9)	7 (53.8)	ND	ND	2 (15.4)
pgD + pCCL19	13	13 (100.0)	8 (61.5)	3 (23.1)	ND	ND	ND	ND
pgD + pCCL28	12	12 (100.0)	7 (58.3)	4 (33.3)	ND	ND	ND	ND
PCCL28-IZ-gD	10	10 (100.0)	9 (90.0)	5 (50.0)	ND	ND	ND	ND

a HSV-2 shedding incidences in vaginal liquids were detected by real-time PCR. Data shown are from 3 or 4 independent experiments. HSV-2 shedding incidence = (number of vaginal liquids from which viral DNA was detected/total number of vaginal liquids analyzed) × 100. —, not obtainable for serious disease. ND, no virus was detected.

### (iv) Latent viral DNA.

As described previously (28), the latent HSV-2 genomes in murine sacral ganglia were quantified at 30 dpi or at the time of death. The immunization groups ranked from highest to lowest number of latent HSV-2 genome copies were pcDNA3.1, pgD plus pcDNA3.1, pgD plus pCCL19, pgD plus pCCL28, and pCCL28-IZ-gD. There were no significant differences between the last three groups, indicating that both CCL28- and CCL19-enhanced Ag-specific responses could prevent HSV-2 from establishing latent infection after lethal challenge (Fig. 6D).

Overall, we initially tested the dose effects of CCL19 and CCL28 to help enhance HSV-2 gD immunogenicity and protect against lethal doses of viral challenge. The results showed that the doses of 20 mol pCCL19 and 10 mol pCCL28, and an even lower dose of a 1:1 ratio of Ag and CCL28 constructs, can protect mice against HSV-2 challenge without weight loss or any apparent symptoms (Table S3). In summary, a high dose of CCL19 is helpful in inducing protective immunity, especially humoral immunity; however, the low dose of CCL28 demonstrated a more potent enhancement of cellular responses in the protective immune response.

## DISCUSSION

A prophylactic HSV-2 vaccine should induce powerful humoral, cellular, and mucosal immunity, which should sterilize HSV-2 shedding and reduce recurrent lesions in the long term (37). Based on our previous study evaluating general chemokines in combination with HIV-1 Env (8), our results show that both CCL19 and CCL28 can augment systemic and mucosal immune responses, while CCL19 can enhance HSV-2 gB-induced protective mucosal immune responses (28). In this study, we systematically compared CCL19 and CCL28 as molecular adjuvants in combination with the vaccine candidate HSV-2 gD to assess whether they promote protective immunity in mice.

It has been reported that mucosa-associated adjuvants codelivered with immunogens can enhance protective immune responses (15, 38, 39). In our study, we found that CCL19 and CCL28 showed dose-dependent differences in their protection against challenge (Fig. S2). Our results also suggest that an effective combination vaccine needs less CCL28 than CCL19. In addition, as for a preventive vaccine against mucosally transmitted viruses such as HSV-2, a strong and rapid mucosal antibody response is required, which can be achieved by a good memory B cell response (40, 41). Compared with CCL19, here we found that CCL28 has an advantage in inducing long-lasting serum neutralizing Abs postchallenge and more potent systemic Abs, which may be the dominant reason for the rapid removal of HSV-2 after challenge and the reduction in establishing latent infection. At the same time, we found that the pgD plus pCCL19 group produced less serum inflammatory cytokines postchallenge, suggesting that the instant recalled neutralizing Abs are more important for viral clearance in the early period of challenge. Previous studies found that CCL19 and HSV-1 gB (15) used in combination could enhance HSV-1 gB-specific systemic IgG and IgA in sera during the memory response. In agreement, our results further demonstrated that CCL19 or CCL28 used in combination with gD or as a fusion construct could significantly enhance the levels of humoral and mucosal protective Abs as well as the duration of protective immune responses compared with gD alone.

However, it has also been concluded from several animal studies that robust humoral immunity alone is ineffective in the control of HSV-2 infection (29, 30). An ideal prophylactic vaccine should not only produce a potent humoral immune response but also promote T cell immune responses (15, 32). To this end, we demonstrated that both CCL19 and CCL28 as adjuvants could promote enhanced gD-specific Th1- and Th2-like cellular responses postimmunization. Previous studies demonstrated that gD-specific IgG2a contributed to a potent neutralizing capacity against HSV-2 in guinea pigs (32). Consistent with some studies (29, 30), our results indicated that HSV-2 gD induces a relatively reduced IgG2a profile, while CCL19 or CCL28 in combination with gD or as a chimeric construct can preferentially induce the IgG2a subtype after immunization and viral challenge; however, codelivery with less CCL28 was more potent than CCL19 in inducing Th1-like IgG2a responses, cytokine-associated Th1/Th2-associated responses, and murine T cell migration to secondary lymph tissues postboost and postchallenge. However, although the pCCL28-IZ-gD chimeric construct produced a Th1-like IgG2a response similar to that by pgD with 20 mol pCCL19, it produced a significantly higher Th1-associated IFN-γ response and higher virus-responsive CCR10^+^ T cell, B cell, and DC immigration rates postchallenge. Taken together, these results indicate that CCL28 is more potent than CCL19 in promoting a higher magnitude and longer duration of Ag-responsive Th1- and Th2-like cellular immunity during viral challenge, implying that a stronger cellular immunity also is important in achieving mucosal immune protection against genital herpes.

Likewise, it is necessary to prove that an appropriate immune response is induced in the mucosal sites to protect against sexually transmitted pathogens. In this study, we developed a DNA construction platform that allows bicistronic and chimeric expression of an Ag and chemokine using IRES and IZ linkers, respectively. In the current study, some linkers may favor enhanced immune responses by facilitating expression of the Ag and adjuvant in the same cell and tissue (27, 28). In addition, as a proof-of-concept study, we adopted a platform for expressing gD and chemokine at a 1:1 ratio to further reveal the working program and efficiency of chemokines *in vivo*, although their optimal doses for triggering appropriate immune responses warrant further investigation. Nevertheless, the CCL28 IZ-based chimeric construct has shown efficacy in priming a robust immune response, particularly a cellular response. Here, the IZ linker demonstrated an advantage over IRES in inducing potent immune responses and protecting mice against viral challenge. We also showed that CCL28 as an adjuvant has a relatively high efficiency in promoting HSV-2 gD-responsive immune protection. Compared to our previous study (28), molecular adjuvant CCL19 combined with HSV-2 gB and IZ linker can produce potent immunity during immune protection rather than CCL28 (data not shown), indicating that required adjuvants vary with different Ags rather than depending on the roles of adjuvants.

In conclusion, our results demonstrate that CCL19 and CCL28 can promote durable, Ag-specific, systematic, and mucosal antibody responses as well as enhanced Th1- and Th2-like cellular responses postimmunization and postchallenge. These enhanced recall responses postchallenge contribute to protecting mice against a lethal dose of HSV-2 challenge. Although future work is warranted to assess the effects of our vaccine candidates in other animal models, findings in this study provide a theoretical basis for vaccine development to exert similar efficacy in humans. Since HSV-2 transmission mostly occurs in localized mucosal sites, the genital herpes model enables us to dissect the roles of different chemokines in recalling mucosa-associated immune responses. Our study suggests that the immune potentiation of these chemokine-formulated vaccines is applicable in pharmaceutical strategies for prophylaxis and in managing human genital herpes.

## MATERIALS AND METHODS

### DNA constructs.

Murine CCL19 and CCL28 chemokine constructs were described previously (8), namely, plasmids pCCL19 and pCCL28. The Ag-truncated gD ectodomain (gD-306aa) was amplified from the HSV-2 G strain genome (LGC Standards) as described previously (42, 43). Constructs of chemokine-Ag (chemokine-IRES-gD, gD-IRES-chemokine, chemokine-IZ-gD, and gD-IZ-chemokine) containing the internal ribosome entry site (IRES) of the encephalomyocarditis virus (EMCV) or IZ linker [(Gly_4_Ser)_2_-IZ-(Gly_4_Ser)_2_] were designed and cloned into pIRES2 (Clontech, USA) or pcDNA3.1(+) (Invitrogen, USA) by following the methods described previously (8). Truncated *gD* was also cloned into the prokaryotic expression plasmid pET28a (Novagen, USA), termed pET-gD. For the IRES-based chemokine-Ag DNA vaccines, both chemokine and the Ag coding gene were accompanied by a stop codon to encode the bicistronic expression constructs. For IZ-based chemokine-Ag DNA vaccines, coding genes placed after the IZ linker had no signal peptides, and a single stop codon was placed at the 3′ end of the IZ[(Gly_4_Ser)_2_-IZ-(Gly_4_Ser)_2_]-linked fusion constructs. For both IRES and IZ designs, we generated constructs with both Ag and chemokines before and after the IRES and IZ sequences, respectively (Fig. 1A; see also Fig. S1A in the supplemental material). Appropriate restriction sites were introduced into the chemokine and Ag coding genes, and they were amplified by splicing overlap extension PCR (27). The PCR primers used in plasmid construction are listed in Tables S1 and S2. The constructs were extracted using the Pro Filter Plasmid Midi/Maxi extraction kit (Macherey-Nagel, Germany) and confirmed by sequencing.

### Cell lines and HSV-2 titer.

Vero and HEK 293T cell lines (ATCC CRL-1573) were cultured in complete Dulbecco’s modified Eagle medium (DMEM) (Invitrogen, USA) supplemented with 10% fetal bovine serum (FBS) (Invitrogen, the Netherlands), 100 U/ml penicillin, and 100 μg/ml streptomycin (Invitrogen, USA).

As described previously (44), the HSV-2 G strain (2.4 × 10^6^ PFU/ml) was propagated on a Vero cell monolayer (multiplicity of infection of 5). At 36 to 48 h, supernatant virus and cell-associated virus (freeze-thawed three times) were harvested and filtered (0.45 μm). Viral stocks were aliquoted and stored at −80°C. The number of PFU of HSV-2 was determined by a plaque assay before use.

### Prokaryotic gD protein expression and purification.

gD protein was produced in Escherichia coli as described previously (28), with modifications. In brief, the E. coli Rosetta strain (Novagen, USA) was first transformed with pET-gD and then induced with isopropyl-β-d-thiogalactoside (IPTG) for 4 h for gD production. Afterwards, the gD protein produced in the bacteria as insoluble inclusion bodies was released by ultrasonic treatment and collected by centrifugation (30 min, 1,200 × *g* at 4°C). The insoluble protein in the lysates was washed twice with phosphate-buffered saline (PBS) plus 1 M guanidine hydrochloride (GuHCl) and subsequently resuspended with denaturing buffer (10 mM Tris, 500 mM NaCl, 6 M GuHCl, and 5 mM dithiothreitol, pH 8.0) over 12 h, followed by refolding in refolding buffer (20 mM Tris-Cl, 500 mM NaCl, 3 mM glutathione, 0.3 mM glutathione disulfide, 10% glycerol, pH 8.0) for 48 h at 4°C (45). Refolded gD protein was purified by nickel-charged Sepharose (GE Healthcare, USA) according to the manufacturer’s instructions. A six-His tag on the C terminus of gD facilitated its purification, followed by elution with imidazole buffer (20 mM Tris-Cl, 500 mM NaCl, 3 mM glutathione, 0.3 mM glutathione disulfide, 300 mM imidazole, 10% glycerol [pH 8]). Subsequently, purified gD protein was dialyzed and aliquoted, and the concentration was determined with a bicinchoninic acid assay kit (Thermo Scientific, USA) before use. The quality of the purified gD protein was also determined by SDS-PAGE and Western blotting (28). Purified products were stored at −80°C.

### Mice, immunizations, and sampling.

Naïve female BALB/c mice (Beijing HFK Biotechnology) aged 6 to 8 weeks were used for plasmid immunization and housed under specific-pathogen-free (SPF) conditions. All experimental procedures involving animals were performed in accordance with the terms approved by the Institutional Animal Care and Use Committee and the Hubei Laboratory Animal Science Association (approval no. WIVA11201301). The prime and boost immunization strategy is shown in Fig. 1B, and the immunization procedure was carried out as described previously (17, 28). In brief, each randomly grouped mouse (*n *=* *10 per group) was injected intramuscularly (i.m.) with various doses of plasmids in a volume of 40 μl into the quadriceps muscles, which was followed by three pulses of electroporation (100 V, 50 ms) and another three pulses with reversed electrode polarity. As shown in Table S3 in the supplemental material, mice were coimmunized with 1 mol gD-encoding plasmid (pgD) plus 1, 10, or 20 mol pCCL19/pCCL28 monovalent DNA plasmid or immunized with 1 mol chimeric DNA construct per mouse (i.e., pCCL19/CCL28-IRES-gD, pgD-IRES-CCL19/CCL28, pCCL19/CCL28-IZ-gD, or pgD-IZ-CCL19/CCL28). For the negative-control group, mice were immunized with an equivalent amount of control vector (pcDNA3.1). As described previously (17), animals were immunized twice at 2-week intervals. Serum and vaginal lavage samples were collected at 2 and 7 weeks postboost. Vaginal lavage samples were collected by washing the vagina three times with PBS containing protease inhibitors (Roche, China). Samples were aliquoted and stored at −80°C until use.

### HSV-2 challenge, scoring, and virus shedding.

Five days prior to HSV-2 challenge, each mouse was injected subcutaneously (s.c.) with medroxyprogesterone acetate (2 mg). At 7 weeks postboost, mice (*n* = 5) were challenged intravaginally (i.vag.) with 10 μl per mouse of HSV-2 (2.4 × 10^7^ PFU/ml) after pentobarbital anesthetization. Weight loss was monitored daily, and the manifestations of vaginal inflammation were scored according to criteria reported by Skoberne et al. (32) and specified from 0 to 5 (0, no apparent inflammation; 1, mild inflammation; 2, redness and moderate swelling; 3, severe redness and inflammation; 4, genital ulceration and severe inflammation; 5, hind-limb paralysis and death). Serum and vaginal lavage samples were collected at the indicated day postinfection (dpi) (Fig. 1B) and used for the detection of Ag-specific IgG titers, viral neutralization Ab titers, Ag-specific inflammatory cytokine responses, and viral shedding. Viral shedding in vaginal lavage samples was tested by plaque-forming assay on Vero cell monolayers as described previously (32, 46). Sacral ganglia were collected at 30 dpi or at the time of mouse death, and then the latent viral load in the ganglia was detected with DNA quantitative fluorescent PCR. HSV-2 DNA extraction and testing were carried out according to a previous study (47). All samples were tested in triplicate.

### Expression analyses of gD and chemokines.

HEK 293T cells were transfected with 0.5 mol pcDNA3.1, pgD plus pcDNA3.1, pgD plus pCCL19, pgD plus pCCL28, and chimeric construct pCCL28-IZ-gD, respectively, for 48 h, and supernatants were harvested and analyzed by 12% denaturing SDS-PAGE and 4 to 15% continuous gradient native PAGE, respectively. The gD glycoprotein moiety in the recombinant proteins was detected by Western blotting, as previously described (28). Anti-gD monoclonal antibody (MAb) (0191; Santa Cruz Biotechnology, USA) and horseradish peroxidase (HRP)-labeled donkey anti-sheep IgG (Santa Cruz Biotechnology, USA) were used to detect gD expression. Chemokines were detected using the CCL19 and CCL28 DuoSet ELISA kit (R&D Systems, USA), respectively.

### Tissue processing and harvesting.

At 2 weeks postboost, spleens and mesenteric lymph nodes (MLNs) were removed under sterile conditions, and a single-cell suspension was obtained by pressing samples through a 70-μm Falcon cell strainer (BD Biosciences, China). Mouse immunocytes were isolated using mouse 1× lymphocyte separation medium according to the manufacturer’s instructions (Dakewe Biotech, China). Finally, red blood cells were lysed by red blood cell lysis buffer (Sigma-Aldrich, USA). Purified immunocytes were resuspended in complete RPMI 1640 medium and counted using an automated cell counter (Bio-Rad, USA).

### Virus neutralization assay.

The neutralization activities of sera and vaginal lavage fluid were tested using plaque assays as described previously, with modifications (2). Briefly, heat-inactivated sera (56°C for 1 h) were prepared in 2-fold serial dilutions from 1:10 to 1:1,280 (postboost) or from 1:40 to 1:5,120 (postchallenge) in DMEM (without FBS). Vaginal lavage fluid was prepared in 2-fold serial dilutions from 1:5 to 1:40 (postboost) in DMEM (without FBS). The diluted samples (in triplicate) were subsequently incubated with 100 PFU/ml of HSV-2 for 1 h at 37°C. Following incubation, the sample-virus mixture was added to Vero cells in 48-well culture plates (preseeded at 1.2 × 10^5^ cells per well 1 day before the assay). Cells receiving HSV-2 alone were included as positive controls, whereas cells without the sample-virus mixture were used as background controls. Sera from pcDNA3.1 immunized mice at the same dilution were considered controls for nonspecific background in inhibiting plaque formation. After incubation for 48 h, cells were stained with crystal violet and the plaques were counted for HSV-2 titration. The Ab-mediated neutralization activity was determined based on the dilution titer that reduced the number of plaques by 50% compared with the positive control and was expressed as the log_10_ of the dilution titer.

### Ag-specific IgG, IgA, and IgG subclasses.

gD-specific IgG and IgA endpoint titers in sera and vaginal lavage fluid were measured by indirect ELISA, as described in our previous study (47). In brief, 96-well microplates (Nunc Maxisorp; Thermo Scientific, USA) were coated with purified gD (2 μg/ml, 50 μl/well) overnight at 4°C and then washed 5 times with wash buffer and blocked with blocking buffer. Thereafter, plates were incubated with serially diluted samples at 37°C for 1 h. Plates were washed 5 times again and incubated with HRP-conjugated goat anti-mouse IgG (Abcam, USA) or biotin-conjugated goat anti-mouse IgA (Southern Biotechnology, USA) at a dilution of 1:5,000 at 37°C for 1 h. For IgA detection, plates were washed 5 times and incubated with streptavidin-HRP (R&D Systems, USA) at a dilution of 1:200 at 37°C for 0.5 h. After 5 washes, bound HRP conjugates were detected by a TMB-based colorimetric reaction at room temperature for 5 min. This reaction was stopped with 2 N H_2_SO_4_. The color intensity was read by an automated ELISA reader (Tecan, Switzerland). Endpoint titers were calculated using GraphPad Prism 8.0. Sample dilutions with an optical density at 450 nm (OD_450_) higher than the OD_450_ of the negative control plus 2× standard deviations (SD) at the same dilution were considered positive.

The titers of gD-specific isotype subclass Abs in immunized and virus-challenged murine sera were measured by HRP-conjugated anti-mouse IgG1, IgG2a, IgG2b, IgG3, and IgM (Southern Biotechnology, USA) according to the manufacturer’s instructions. Antibody concentrations were calculated based on standard curves.

### Ag-specific inflammatory cytokine responses.

Postchallenge splenocytes (1 × 10^7^/well) were added to duplicate wells in 24-well plates in a total volume of 1 ml complete RPMI 1640 with purified gD protein (2 μg/ml) as a stimulus and cultured at 37°C for 5 days. After incubation, cell culture supernatants were filtered and used for the detection of Th1/Th2 cytokines (IL-2, IL-4, IL-5, IFN-γ, and TNF-α). In addition, antisera were collected at 5 and 9 dpi and used for the detection of Th1/Th2 cytokines. The analysis was performed with a BD cytometric bead array mouse Th1/Th2 cytokine kit (BD Biosciences, China) according to the manufacturer’s instructions. Data acquisition was conducted on a BD FACSAria III platform and analyzed by FCAP Array software 2.0.

### Immunohistochemistry of rectal mucosae.

At 2 weeks postboost, murine colorectal samples were collected and analyzed for IgA^+^ cell number by immunohistochemistry in accordance with the procedures described previously, with some modifications (8, 20). In brief, an approximately 2-cm segment from the end of the anus of each mouse was isolated, cut open longitudinally, and fixed in neutral buffered formalin (diluted 1:10 in PBS) for 24 h. After fixation, tissue was embedded and sectioned. Ag retrieval was performed with the Ag retrieval reagent-basic (R&D Systems, USA) according to the manufacturer’s instructions. For the detection of IgA^+^ cells, the slides were first stained with goat anti-mouse IgA antibody (AbD Serotec, UK) at a dilution of 1:400 overnight at 4°C, followed by incubation with the Polink-2 plus polymer HRP detection system for goat primary antibody (GBI, China) according to the manufacturer’s instructions. A color reaction was developed with the addition of 3,3′-diaminobenzidine free base (DBA), followed by counterstaining with hematoxylin.

### Chemokine-specific chemotaxis reactions.

Chemotaxis assays with purified splenocytes and MLN lymphocytes (MLNLs) were performed as previously described (28). A microchamber Transwell system with 3-μm pores (Corning Costar, USA) was used in the chemotaxis assays. In brief, 2 × 10^6^ cells/well (in 300 μl complete RPMI 1640 medium) were plated in triplicate in the upper chambers, and 600 μl complete RPMI 1640 medium with or without (negative control) 30 ng/ml CCL19 or 500 ng/ml CCL28 (R&D Systems, USA) was added into the lower chambers. The plates were incubated at 37°C for 90 min, and the number of migrated cells was counted using a Bio-Rad automated cell counter. The fold change in migration was calculated as migrated cell number in testing wells divided by migrated cell number in negative-control wells.

### Statistical analysis.

Comparisons between two groups were done using the nonparametric *t* test, while more than three groups were analyzed by two-way analysis of variance with Dunnett’s correction analysis (GraphPad Prism 8.0). Data are presented as the means ± standard errors of the means (SEM). A *P* value of <0.05 was considered statistically significant.
