# Supplementary material for: CCL19 and CCL28 Assist Herpes Simplex Virus 2 Glycoprotein D To Induce Protective Systemic Immunity against Genital Viral Challenge
Source: mSphere. 2021 Apr 28;6(2):e00058-21. doi: 10.1128/mSphere.00058-21 (PMC8092132; doi:10.1128/mSphere.00058-21)
Supplement: TABLE S1 [file mSphere.00058-21-st001.docx]

**Table S1 Sequences of primers and digestion sites of “chemokine-IRES-gD” and “gD-IRES-chemokine” bicistronic expressing constructs**

| **Sequences name** | **Primer (5'→3')** | **Fragment length**  **(bp)** |
| --- | --- | --- |
| **IRES2-EcoR I** | F: TATGAATTCGCCCCTCTCCCTCCCCCCCC | 606 |
| **IRES2-Xho I** | R: CCGCTCGAGGGTTGTGGCCATATTATCAT |  |
| **CCL19-BamH I** | F: TATAGGATCCATGGCCCCCCGTGTGACCCC | 347 |
| **CCL19-EcoR I** | R: CCCGGAATTCTCAAGACACAGGGCTCCTTC |  |
| **CCL28-BamH I** | F: TACGGATCCATGCAGCAAGCAGGGCTCAC | 411 |
| **CCL28-EcoR I** | R: CCGGAATTCCTAACGAGAGGCTTCGTGCC |  |
| **gD** **-Xba I** | F: GCTGCTCTAGAATGGGGCGTTTGACCTC | 918 |
| \| **gD-Pme I** \|  \| \| --- \| --- \| | R: GCGTTTAAACCTAGTAAAACAATGGCTG |  |
| **gD- BamH I** | F: TATAGGATCCATGGGGCGTTTGACCTC | 918 |
| \| **gD-EcoR I** \|  \| \| --- \| --- \| | R: CCCGGAATTCTCTAGTAAAACAATGGCTG |  |
| **CCL19- Xba I** | F: GCTGCTCTAGAATGGCCCCCCGTGTGACCCC | 347 |
| **CCL19- Pme I** | R: GCGTTTAAACTCAAGACACAGGGCTCCTTC |  |
| **CCL28- Xba I** | F: GCTGCTCTAGAATGCAGCAAGCAGGGCTCAC | 411 |
| **CCL28- Pme I** | R: GCGTTTAAACCTAACGAGAGGCTTCGTGC |  |
