# Supplementary material for: CCL19 and CCL28 Assist Herpes Simplex Virus 2 Glycoprotein D To Induce Protective Systemic Immunity against Genital Viral Challenge
Source: mSphere. 2021 Apr 28;6(2):e00058-21. doi: 10.1128/mSphere.00058-21 (PMC8092132; doi:10.1128/mSphere.00058-21)
Supplement: TABLE S2 [file mSphere.00058-21-st002.docx]

**Table S2** **Sequences of primers and digestion sites of “chemokine-IZ-gD” and “gD-IZ-chemokine” fusion constructions**

| **Sequences name** | **Primer (5'→3')** | **Fragment length (bp)** |
| --- | --- | --- |
| **CCL19-Hind III** | F: CCC AAG CTT ATG GCC CCC CGT GTG AC | 327 |
| **CCL19-Kpn I** | R: CGG GGT ACC AGA CAC AGG GCT CCT TC |  |
| **CCL28-Hind III** | F: CCC AAG CTT ATG CAG CAA GCA GGG CTC AC | 393 |
| **CCL28-Kpn I** | R: CGG GGT ACC ACG AGA GGC TTC GTG CCT GT |  |
| **CCL19/ CCL28-IZ1** | F: ATC GCC AGA ATC AAG AAG CTG ATC GGC GAG AGA GGT GGC GGT GGC TCC GGC GG |  |
| **CCL19/ CCL28-IZ2** | F: ATC CTG AGC AAG ATC TAC CAC ATC GAG AAC GAG ATC GCC AGA ATC AAG AAG CT |  |
| **CCL19/ CCL28-IZ3- BamH I** | F: TAT GGA TCC AGA ATG AAG CAG ATC GAG GAC AAG  ATC GAG GAG ATC CTG AGC AAG ATC TAC CA |  |
| **IZ4-(G4S)2(EcoR I)** | F: CCG GAA TTC AGA ATG AAG CAG ATC GAG GA |  |
| **gD-EcoR I** | F: CCG GAA TTC GCC AAA TAC GCC TTA GCA G | 924 |
| **gD-Xba I** | R: TGC TCT AGA ATC CTC TAA GAG GGC CG |  |
| **gD- Hind Ⅲ** | F: CCC AAG CTT ATG GGG CGT TTG ACC TCC GG | 1055 |
| \| **gD-(G4S)2-EcoR I** \|  \| \| --- \| --- \| | R: CCG GAA TTC GGT GGC GGT GGC TCC GGC GGT GTG GGT CCA TCC TCT AAG AGG GCC G |  |
| **CCL19-(G4S)2-BamH I** | F: TAT GGA TCC GGT GGC GGT GGC TCC GGC GGT GGT GGG TCC GGT GCT AAT GAT GCG GAA GA | 300 |
| **CCL19-Xho I** | R: CCG CTC GAG TCA AGA CAC AGG GCT CCT TC |  |
| **CCL28-(G4S)2-BamH I** | F: TAT GGA TCC GGT GGC GGT GGC TCC GGC GGT GGT GGG TCC ATA CTT CCA TGG CCT CCA G | 375 |
| **CCL28-Xho I** | R: GGC CTC GAG CTA ACG AGA GGC TTC GTG CC |  |
