# Supplementary material for: CCL19 and CCL28 Assist Herpes Simplex Virus 2 Glycoprotein D To Induce Protective Systemic Immunity against Genital Viral Challenge
Source: mSphere. 2021 Apr 28;6(2):e00058-21. doi: 10.1128/mSphere.00058-21 (PMC8092132; doi:10.1128/mSphere.00058-21)
Supplement: TABLE S3 [file mSphere.00058-21-st003.docx]

**Table S3** **HSV-2 challenge and score (mean)**

| Group | Total animal  number | Average scores of mice challenged with HSV-2 | | | | | | |  |
| --- | --- | --- | --- | --- | --- | --- | --- | --- | --- |
|  |  | 1 dpi | 3 dpi | 5 dpi | 7 dpi | 9 dpi | 11 dpi | 15 dpi | 30 dpi |
| pcDNA3.1 (1, 10, 20 mol) | 14 | 0 | 1.9 | 4.3 | - | - | - | - | - |
| pgD (1 mol) + pcDNA3.1(1, 10, 20 mol) | 13 | 0 | 0.2 | 0.5 | 0.5 | 0.6 | 0.7 | 0.4 | 0.4 |
| pgD (1 mol) + pCCL19 (1 mol) | 10 | 0 | 0.1 | 0 | 0 | 0 | 0 | 0 | 0.2 |
| pgD (1 mol) + pCCL19 (10 mol) | 10 | 0 | 0 | 0 | 0 | 0 | 0 | 0 | 0 |
| pgD (1 mol) + pCCL19 (20 mol) | 10 | 0 | 0 | 0 | 0 | 0 | 0 | 0 | 0 |
| pCCL19-IRES-gD (1 mol) | 10 | 0 | 0.3 | 0.6 | 2.3 | 3.6 | 4.3 | 4.5 | 5 |
| pgD-IRES-CCL19 (1 mol) | 10 | 0 | 0.2 | 0.5 | 1.2 | 2 | 4 | 3 | 3 |
| pCCL19-IZ-gD (1 mol) | 10 | 0 | 0.8 | 1.1 | 2.4 | 3.2 | 4.2 | 4.8 | 4.9 |
| pgD-IZ-CCL19 (1 mol) | 13 | 0 | 0 | 0.2 | 0.3 | 0.4 | 1 | 1 | 0.8 |
| pgD (1 mol) + pCCL28 (1 mol) | 10 | 0 | 0 | 0 | 0 | 0 | 0 | 0 | 0 |
| pgD (1 mol) + pCCL28 (10 mol) | 10 | 0 | 0 | 0 | 0 | 0 | 0 | 0 | 0 |
| pgD (1 mol) + pCCL28 (20 mol) | 10 | 0 | 0 | 0 | 0 | 0 | 0 | 0 | 0 |
| pCCL28-IRES-gD (1 mol) | 12 | 0 | 0.1 | 1.5 | 2.3 | 2.7 | 2.7 | 2.8 | 3.0 |
| pgD-IRES-CCL28 (1 mol) | 10 | 0 | 1.8 | 3.6 | 4.1 | 4.4 | 5 | - | - |
| pCCL28-IZ-gD (1 mol) | 10 | 0 | 0 | 0 | 0 | 0 | 0 | 0 | 0 |
| pgD-IZ-CCL28 (1 mol) | 10 | 0 | 0 | 0.1 | 0.3 | 0.4 | 0.7 | 0.9 | 1.2 |

Scores: 0, no apparent inflammation; 1, mild inflammation；2, redness and moderate swelling; 3, severe redness and inflammation; 4, genital ulceration and severe inflammation; 5, hind limb paralysis and death. Data shown are from 2 independent experiments; dpi: days post infection; -, Death.
