# Supplementary figures and images for: CCL19 and CCL28 Assist Herpes Simplex Virus 2 Glycoprotein D To Induce Protective Systemic Immunity against Genital Viral Challenge
Source: mSphere. 2021 Apr 28;6(2):e00058-21. doi: 10.1128/mSphere.00058-21 (PMC8092132; doi:10.1128/mSphere.00058-21)

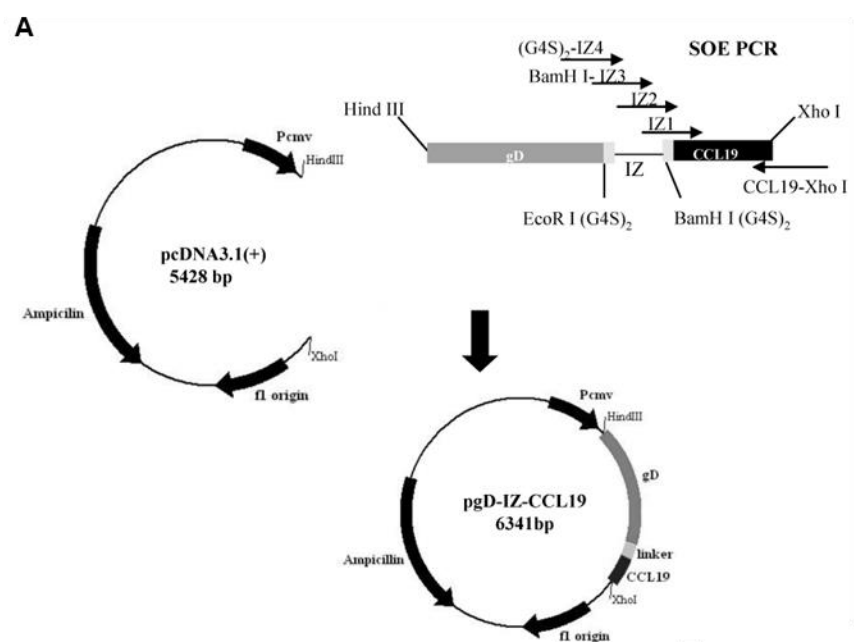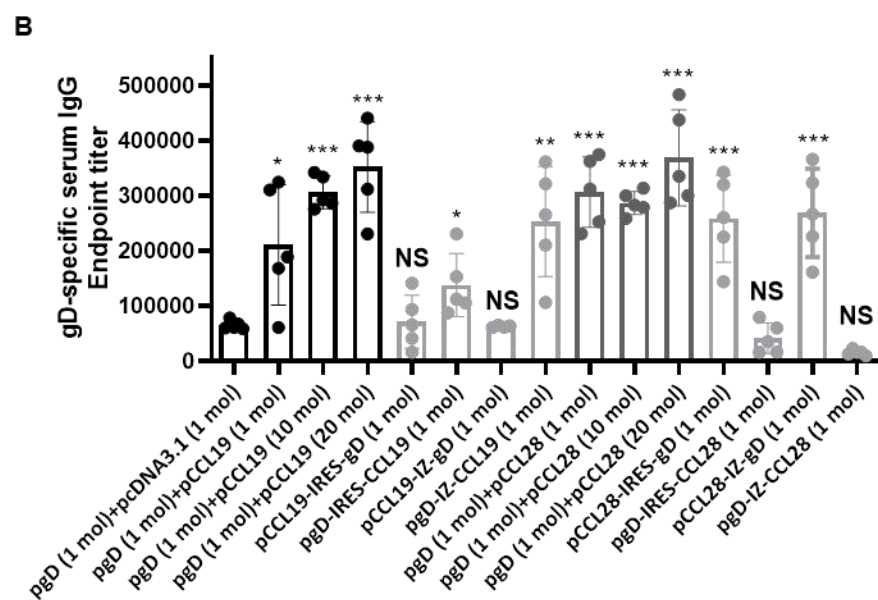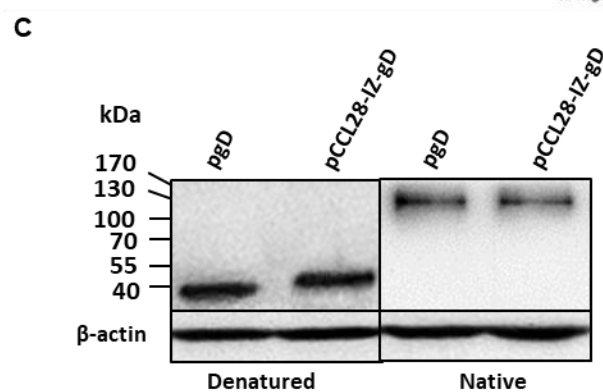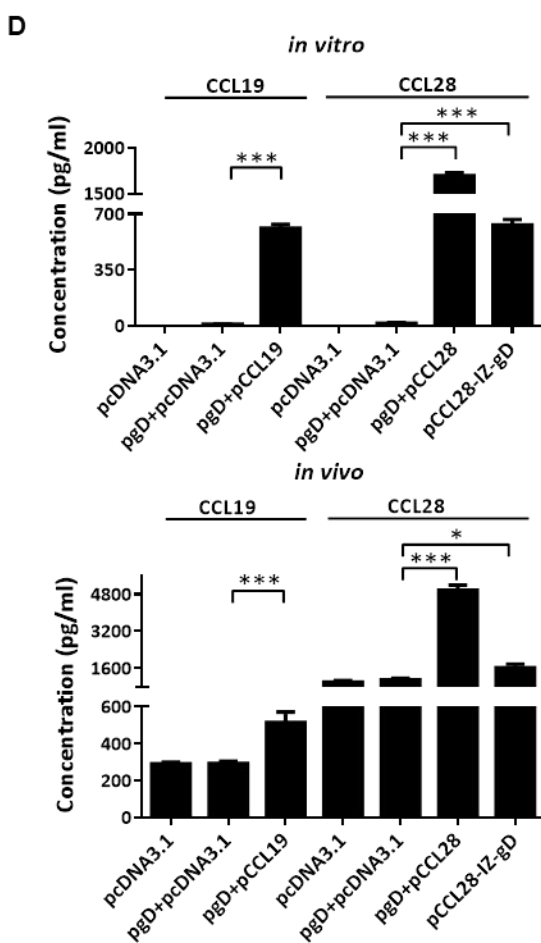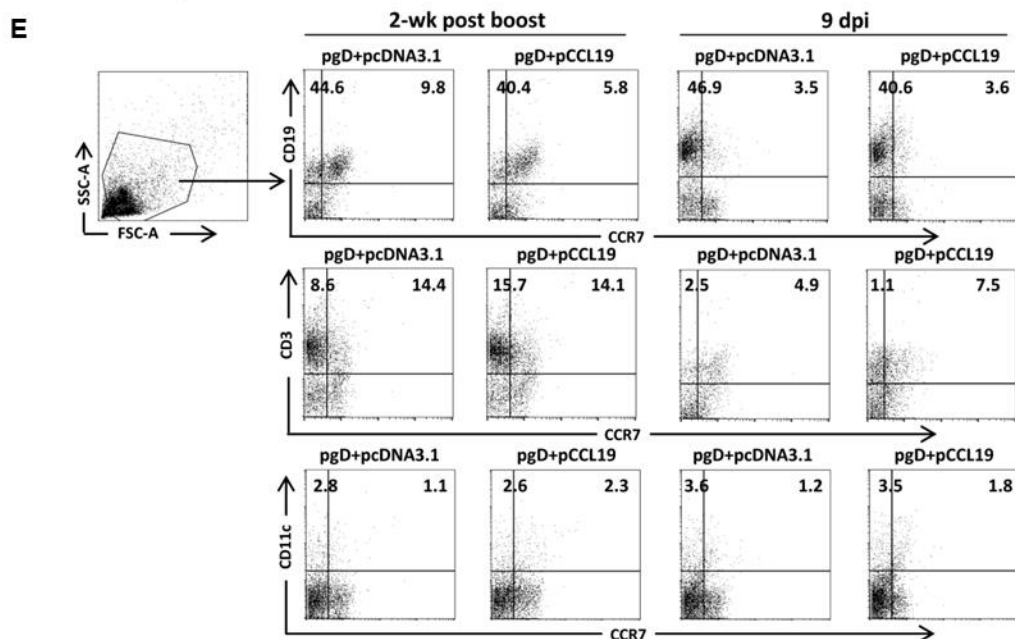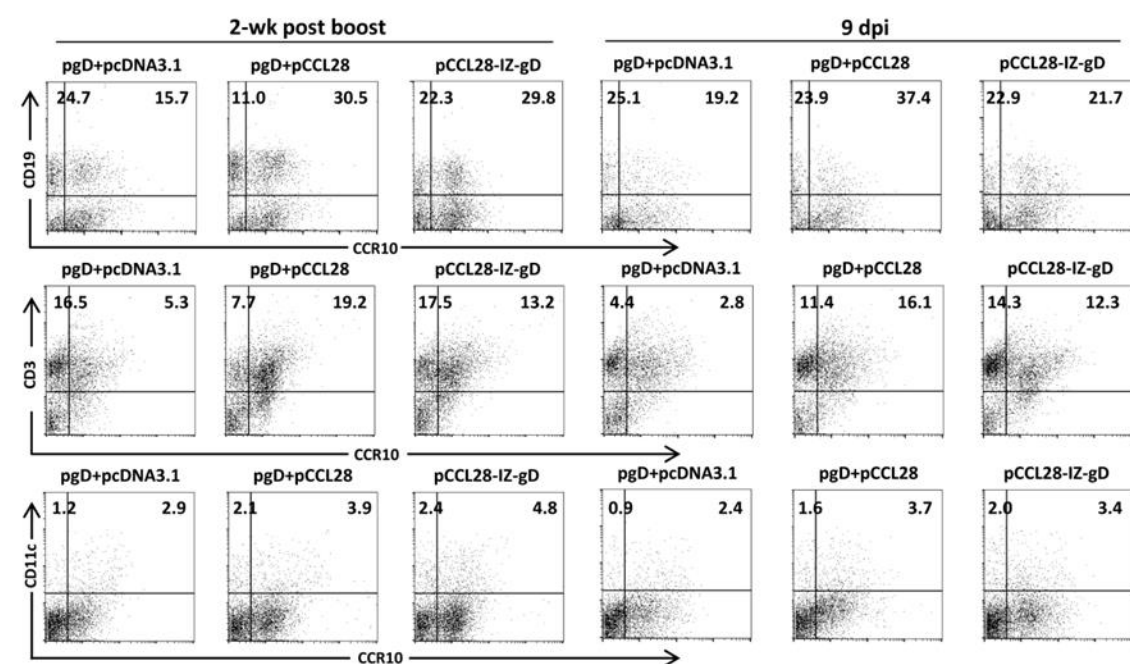

Supplement: FIG S1 [file mSphere.00058-21-sf001.pdf]
